# Oncological outcomes, quality of life outcomes and complications of partial cystectomy for selected cases of muscle-invasive bladder cancer

**DOI:** 10.1038/s41598-018-26089-x

**Published:** 2018-05-30

**Authors:** Jan Ebbing, Robin Colja Heckmann, Justin William Collins, Kurt Miller, Barbara Erber, Frank Friedersdorff, Tom Florian Fuller, Jonas Busch, Hans Helge Seifert, Peter Ardelt, Christian Wetterauer, Abolfazl Hosseini, Florian Jentzmik, Carsten Kempkensteffen

**Affiliations:** 1 University Hospital Basel, Urological University Clinic Basel-Liestal, Spitalstrasse 21, 4056 Basel, Switzerland; 2 Charité - University Hospital, Department of Urology, Chariteplatz 1, 10117 Berlin, Germany; 3 Karolinska Institutet, Department of Molecular Medicine and Surgery (MMK), Karolinska University Hospital, Solna, 171 76 Stockholm, Sweden; 4 Vivantes Hospital Am Urban, Department of Urology, Dieffenbachstraße 1, 10967 Berlin, Germany; 5 Karolinska University Hospital, Department of Urology, Solna, 171 76 Stockholm, Sweden; 6 Oberschwaben Clinic, Hospital St. Elisabeth, Department of Urology, Elisabethenstr. 15, 88212 Ravensburg, Germany; 7 Franziskus Hospital Berlin, Department of Urology, Budapester Strasse 15-19, 10787 Berlin, Germany

## Abstract

To evaluate the oncological results, associated complications, and postoperative health-related quality of life (HR-QoL) in patients treated with partial cystectomy (PC) for muscle-invasive bladder cancer (MIBC). 27 patients who underwent open PC for cT2 MIBC were included. A simple Cox’s proportional hazards regression model was used to assess the association of several potential prognostic factors with survival. Postoperative HR-QoL was assessed with the EORTC (European Organisation for the Research and Treatment of Cancer) QLQ-C30 questionnaire version 3.0. Final pathological tumour stages in PC specimen were: pT0: 18.5%, non-MIBC: 3.7%, MIBC: 74.1%, pCIS: 14.8%. Estimated 5-year overall- and progression-free survival rates were 53.7% and 62.1%. Five (18.5%) patients experienced local recurrence with MIBC. Overall, the salvage cystectomy rate was 18.5%. The 90-day mortality rate was 0%. Significant risk factors for progression-free survival were vascular invasion (HR 5.33) and tumour multilocularity (HR 4.5) in the PC specimen, and a ureteric reimplantation during PC (HR 4.53). The rates of intraoperative complications, 30- and 90-day major complications were 7.4%, respectively and 14.8% for overall long-term complications. Postoperatively, median (IQR) global health status and QoL in our PC cohort was 79.2 (52.1–97.9). Open PC can provide adequate cancer control of MIBC with good HR-QoL in highly selected cases. Open PC can lead to long-term bladder preservation and shows an acceptable rate of severe perioperative complications, even in highly comorbid patients.

## Introduction

Bladder cancer (BC) is one of the most common cancers, accounting for 3% of all malignant tumours^1^. Radical cystectomy (RC) is considered to be the accepted gold standard treatment for patients with localized (T2-T4a) muscle-invasive bladder cancer (MIBC) or recurrent high-risk non-MIBC^2^.

However, it is recognised that RC is associated with side effects such as erectile dysfunction and urinary incontinence, and also with considerable morbidity. Complications related to RC were found to be directly related to age, pre-existing comorbidities, as well as to the surgical procedure, bowel anastomosis, or urinary diversion^3–5^.

Hence, alternative therapeutic options for high-risk patients, like organ-sparing partial cystectomy (PC) or radiochemotherapy following radical transurethral resection of the bladder tumour (TURBT), have gained attention. Accordingly, the EAU (European Association of Urology) guidelines do not provide definitive recommendations, but argue that the decision regarding bladder-sparing or radical cystectomy in elderly patients with invasive bladder cancer should be based on tumour stage and comorbidity^2^.

Over the past decade, a growing body of literature has suggested PC to represent a feasible alternative with RC-comparable oncological results in selected cases, associated with lower complication rates, and thus promoting further discussion about the standard-of-care options for selected patients^6–10^. Thereby, selection criteria for PC are a subject of ongoing debate. Suitable candidates typically have a solitary tumour in an area accessible for organ-sparing surgery with absence of carcinoma *in situ* (CIS). For these patients, and especially for those with comorbidities, PC represents a potentially less morbid operation that maintains an intact bladder with normal voiding function, avoids opening of the intestinal tract, and preserves sexual function.

However, data on PC in MIBC patients, especially regarding perioperative complication rates and postoperative health-related quality of life (HR-QoL), is still limited and generally based upon small cohort studies. This work aims to report a single-center’s experience in terms of oncological outcomes, postoperative HR-QoL, and perioperative morbidity in selected T2 MIBC patients who underwent PC with curative intent. To our knowledge, this is the first study reporting Clavien-Dindo classification for the description of early and late postoperative complications and HR-QoL data in an open PC series. In addition, a unique attribute of this patient cohort is that PC was performed in many patients that had tumour locations that are conventionally regarded as contra-indicated for PC.

## Methods

### Patient population and indication for PC

Data collection was performed and written informed consent obtained in accordance with the requirements of the local ethics committee of the Charité - Universitätsmedizin Berlin. Of 35 identified patients that were treated with PC for cT2 cM0 MIBC at the Department of Urology of the Charité University Hospital Berlin between 1995 and 2012, we used our prospectively populated database to retrospectively include 27 patients with complete follow-up data. Patients that were treated by means of PC were very selectively chosen: indication for PC was mainly based on patients’ request for bladder preservation for various reasons, including fear of quality of life impairment related to incontinence or urinary diversion, loss of sexual function, morbidity, and complications. Furthermore, selected patients with severe comorbidities, thus not suitable for RC, and/or patients that refused to obtain (chemo-)radiotherapy, were offered a PC. Every patient was fully informed about the advantages and disadvantages of RC and PC or further alternative options like radiochemotherapy. Neoadjuvant chemotherapy (NAC) was offered to suitable candidates. All patients were staged by computed tomography (CT) or magnetic resonance imaging (MRI) before PC. Diagnostic cystoscopy and transurethral resection of the index tumour with histopathological proof of muscle-invasion was performed in every patient prior to PC. Five patients had been previously treated with Bacillus Calmette-Guerin (BCG) intravesical instillation therapy in their bladder cancer management, four patients due to CIS and one patient due to pT1 high-grade urothelial cancer of the bladder. At the discretion of the treating physician, random biopsies were performed in 41.2% of the patients before PC and in 100% of the patients with prior history of CIS.

Exclusion criteria for PC were persisting CIS after BCG intravesical therapy, local tumour extension with a 1–2 cm safety margin not feasible for PC, and a low-capacity or malfunctioning bladder. Multilocularity of the tumour and the need for ureter reimplantation were not considered as contraindications. Patients with urachal tumours, non-MIBCs, and tumours in bladder diverticula were not included in this study.

### Outcome measures

Surgery related complications: Postoperative complications were classified according to Clavien-Dindo^11^. A score ≥3 was considered a major complication. Early and late postoperative complications were defined as complications before or after 90 days following PC.

Oncological outcomes: Patients were followed according to the EAU guidelines for patients with MIBC following RC, with some risk-adapted modifications in selected individuals. Additionally, patients underwent cystoscopy and urinary cytology at 3 months post PC and subsequently every 3 months for a period of 2 years, and every 6 months thereafter until 5 years, and then yearly following the EAU guidelines for non MIBCs with high risk of recurrence and progression^12^.

Survival rates: Overall survival (OS; time between PC and last follow-up or death), cancer-specific survival (CSS; time between PC and BC related death), progression-free survival (PFS; time between PC and first tumour progression, defined as local MIBC recurrence and/or metachronous metastasis and/or BC related death), local recurrence-free survival (RFS; time between PC and first local recurrence of BC, irrespective of whether superficial urothelial cancer [non-MIBC ≈ pTa, pT1, pCis] or MIBC), local MIBC-recurrence-free survival (MRFS; time between PC and first local recurrence of MIBC), and metastasis-free survival (MFS; time between PC and first detection of a metachronous metastasis) rates were assessed.

Health-related quality of life (HR-QoL) outcomes: Postoperative HR-QoL was assessed with the cancer-specific EORTC (European Organisation for the Research and Treatment of Cancer) QLQ-C30 questionnaire version 3.0^13^ in 12 patients who completed the questionnaire independently at a median of 4.6 (2.8–7.7) years postoperatively. The QLQ-C30 questionnaires were analysed according to the instructions in the EORTC scoring manual^14^. HR-QoL data of the 12 PC patients were contrasted with EORTC-QLQ-C30 questionnaire data of 58 patients treated with RC and urinary diversion (ileal conduit: n = 24, orthotopic ileal neobladder: n = 34) at the Charité between 1993 and 2007, as published previously^15^.

Additionally, following data was assessed for each patient: patient demographics, prior history of BC management, tumour characteristics of the PC specimen, surgical management, adjuvant therapies, and the management of tumour recurrence and/or progression. All patients were staged according to the 2009 TNM system.

### Statistical analyses

The statistical analyses were performed with IBM’s SPSS Statistics, version 23.0 (SPSS Inc., Chicago, Illinois, USA). Continuous data is presented as median and interquartile range (IQR). HR-QoL data is presented as mean with standard deviation (SD). An independent samples t-test analysis (in case of normally distributed data) and a Mann-Whitney U-test of differences (in case non-normally distributed data) were used to compare differences between HR-QoL outcomes of the 12 PC-patients in this study and the 58 RC-patients in a previously published study^15^. The 5-year survival rates for OS, CSS, PFS, RFS, MRFS, and MFS were estimated using Kaplan-Meier analysis. Simple Cox’s proportional hazards regression model was used to test the significance of several potential prognostic factors associated with OS, CSS, PFS, and RFS. Due to the limited number of events, a multivariate regression model was not meaningful. All tests were performed at a significance level of α = 0.05.

### Surgical technique

Surgery was performed with an exptraperitoneal technique, as previsously described^16^. Pelvic lymphadenectomy (PLND) was carried out including the external iliac nodes, the obturatoric nodes, the internal iliac nodes, and the presacral nodes^17^. The bladder tumours were removed with a 1–2 cm safety margin, which resulted in ureteric reimplantation in some cases. Negative surgical margins were confirmed by separate intraoperative frozen sections of the resection margin or by intraoperative assessment of the full PC specimen.

### Data availability

The datasets generated and analysed during the current study are not publicly available due to relevant data protection formalities but are available from the corresponding author J.E. on reasonable request.

## Results

Table 1 lists patient characteristics and comorbidities (Table 1A), prior histories of urothelial carcinoma (Table 1B), tumour characteristics of the PC specimen (Table 1C), surgical characteristics (Table 1D), and hospitalization details (Table 1E). Significant comorbidities were identified throughout the cohort. Overall, 63.0% of the patients had a Charlson comorbidity grade ≥3, and 48.1% were considered severe comorbidity in the Adult Comorbidity Evaluation-27 (ACE-27) score (Table 1A). No patient received NAC due to contraindications or patients’ lack of willingness. The tumour stage of the PC specimen showed pathological up-staging to pT3 disease in 12 (44.1%) patients and down-staging in 6 (22.2%) patients, with 5 (18.5%) patients being staged as pT0. Four (14.8%) patients had CIS on histology of the PC specimen (Table 1C). A median number of 8 (3–10) lymph nodes were identified with unilateral PLND, performed on the tumour side in 8 (29.6%) cases, and 12 (9–17) lymph nodes when bilateral PLND was performed in 17 (63.0%) cases (Table 1D). Overall, 6 (22.2%) patients were found to have regional lymph node metastases. One (3.7%) patient suffered from an incidental subcutaneous metastasis in the abdominal wall, which was treated with local resection during PC. A transperitoneal approach to PC was only necessary in 1 (3.7%) patient. The most common tumour locations were the lateral walls (48.1%), the bladder dome (37.0%), and the posterior wall (22.2%). Overall, 9 (33.3%) tumours were located close to the ureteric orifices, requiring ureter reimplantation in all 9 cases to remove tumour entirely. The median maximum tumour diameter was 30 (20–46) mm, with 59.3% of the tumours being solitary and 40.7% multilocular (Table 1C). Surgical and hospitalization details are summarized in Table 1D and E.

**Table 1:** (**A**) Patient characteristics and comorbidities, (**B**) prior history of urothelial carcinoma of the bladder, (**C**) tumour characteristics of the PC specimen, (**D**) surgical characteristics, and (**E**) hospitalization course.

	Overall cohort (n = 27)
(**A**)	
**Epidemiological characteristics**	
**Age (**years)	66.0 (56.0–76.0)
**Sex** m/f (%)	19/8 (70.4/29.6)
**Plasma creatinine** (mg/dl)	0.85 (0.74–1.12)
**Haemoglobin** (g/dl)	13.8 (12.8–15.2)
**Charlson comorbidity index**	3 (0–5)
**Charlson comorbidity grade** (age adjusted) (%)	
Median grade	3 (2–4)
Grade 1 (no comorbidity) = o pts	2 (7.4)
Grade 2 (slight-moderate comorbidity) = 1–2 pts	8 (29.6)
Grade 3 (intermediate-severe comorbidity) = 3–4 pts	5 (18.5)
Grade 4 (very severe comorbidity) ≥5 pts	12 (44.4)
**Adult Comorbidity Evaluation-27** (ACE-27) (%)	
None	6 (22.2)
Mild	5 (18.5)
Moderate	3 (11.1)
Severe	13 (48.1)
**Karnofsky index (%)**	
100%	16 (59.3)
90%	2 (7.4)
80%	6 (22.2)
70%	2 (7.4)
60%	1 (3.7)
0–50%	0 (0.0)
**ECOG performance status (%)**	
1	18 (66.7)
2	8 (29.6)
3	1 (3.7)
4–5	0 (0.0)
(**B**)	
**Prior history of urothelial carcinoma of the bladder**	
**Primary pathological tumour stage (UICC 2009)** (%)	
pTa	2 (7.4)
pT1	4 (14.8)
pT2	21 (77.8)
pT2a	4 (14.8)
pT2X	17 (63.0)
**Presence of concomitant Carcinoma** ***in situ*** **(CIS)** (%)	4 (14.8)
**Primary tumour grading (WHO 1973)** (%)	
Grade 1	2 (7.4)
Grade 2	4 (14.8)
Grade 3	21 (77.8)
**Primary tumour grading (WHO 2004)** (%)	
High-grade papillary urothelial carcinoma	25 (92.6)
Low-grade papillary urothelial carcinoma	2 (7.4)
**Tumour stage at time of indicaton for PC (UICC 2009)** (%)	
p/cT2	27 (100.0%)
pT2a	4 (14.8)
pT2X	23 (85.2)
**Presence of concomitant Carcinoma** ***in situ*** **(CIS) at time of indication for PC** (%)	0 (0.0)
**Tumour grading at time of indication for PC (WHO 1973)** (%)	
Grade 1	0 (0.0)
Grade 2	3 (11.1)
Grade 3	24 (88.9)
**Tumour grading at time of indication for PC (WHO 2004)** (%)	
High-grade papillary urothelial carcinoma	27 (100.0)
**Neoadjuvant therapy prior PC** (%)	5 (18.5)
BCG intravesical instillation	5 (18.5)
Mitomycin intravesical instillation	0 (0.0)
Chemotherapy	0 (0.0)
Radiation	0 (0.0)
**Time to progression from non-MIBC to MIBC (pT2) prior PC** (months)	9.2 (1.8–40.7)
(**C**)	
**Tumour characteristics of the PC specimen**	
**Histology of the PC specimen (%)**	
Pure urothelial carcinoma	18 (66.7)
Urothelial carcinoma + mixed histology	2 (7.4)
Pure squamous cell carcinoma	2 (7.4)
Pure adenocarcinoma	0 (0.0)
No malignancy	5 (18.5)
**Maximum tumour diameter** (mm)	30 (20–46)
**Tumour location** (%)	
Solitary	16 (59.3)
Multilocular	11 (40.7)
**Number of tumour areas (%)**	
1	16 (59.3)
2	10 (37.0)
3	1 (3.7)
**Location of tumour areas (%)**	
Bladder dome	10 (37.0)
Right side wall	6 (22.2)
Left side wall	7 (25.9)
Bladder ground	3 (11.1)
Anterior wall	1 (3.7)
Posterior wall	6 (22.2)
Right ostium	2 (7.4)
Left ostium	2 (7.4)
Bladder neck	1 (3.7)
Unknown	1 (3.7)
**Tumour stage TNM (UICC 2009)**	
**Pathological tumour stage T (%)**	
pT0	5 (18.5)
pTa	0 (0.0)
pT1	1 (3.7)
pT2	8 (29.6)
pT2a	2 (7.4)
pT2b	4 (14.8)
pT2X	2 (7.4)
pT3	12 (44.4)
pT3a	6 (22.2)
pT3b	5 (18.5)
pT3X	1 (3.7)
pT4	0 (0.0)
pTis (only)	1 (3.7)
**Presence of concomitant Carcinoma** ***in situ*** **(CIS) (%)**	3 (11.1)
**Regional lymph node status N** (%)	
pN0	19 (70.4)
pN1	4 (14.8)
pN2	2 (7.4)
pN3	0 (0.0)
pN+	6 (22.2)
pNX	2 (7.4)
Number of positiv lymph nodes (metastasis)	1.0 (1.0–3.0)
**Distant metastasis M** (%)	
M0	26 (96.3)
M1	1 (3.7)
MX	0 (0.0)
**Location of distant metastasis** (%)	
Subcutaneous	1 (3.7)
**Grading (WHO 1973)** (%)	
Grade 1	0 (0.0)
Grade 2	0 (0.0)
Grade 3	21 (77.8)
Unknown	6 (22.2)
**Grading (WHO 2004)** (%)	
High-grade papillary urothelial carcinoma	21 (77.8)
Low-grade papillary urothelial carcinoma	0 (2.9)
Unknown	6 (22.2)
**Lymphovascular invasion** (%)	
L0	21 (77.8)
L1	5 (18.5)
LX	1 (3.7)
**Vascular invasion** (%)	
V0	19 (70.4)
V1	7 (25.9)
VX	1 (3.7)
**Surgical margins** (%)	
R0	22 (81.5)
R1	4 (14.8)
R2	0 (0.0)
RX	1 (3.7)
(**D**)	
**Surgical characteristics**	
**Operative time** (min)	143.0 (114.0–220.0)
**Pelvic lymph node dissection** (%)	
Yes	25 (92.6)
No	2 (7.4)
**Extent of pelvic lymph node dissection** (%)	
Unilateral	8 (29.6)
Bilateral	17 (63.0)
Number of lymph nodes dissected	10.0 (8.0–15.0)
**Ureter reimplantation** (%)	9 (33.3)
Right side/left side	6 (22.2)/3 (11.1)
**Intraoperative ureter stenting** (%)	12 (44.4)
(**E**)	
**Hospitalization course**	
**Hospitalization time** (days)	10.0 (10.0–15.0)
**Intensive/Intermediate Care Unit rate** (%)	10.0 (37.0)
**Intensive/Intermediate Care days**	1.0 (1.0–4.0)
**Removal of bladder catheter** (p.o. day)	8.0 (7.0–10.0)
**Removal of ureter stent** (p.o. day)	13.0 (10.0–32.0)

Continuous data are shown as median with interquartile range (IQR). (PC) partial cystectomy, (m) male, (f) female, (ECOG) Eastern Cooperative Oncology Group, (UICC) Union Internationale Contre le Cancer, (MIBC) muscle-invasive bladder cancer, (p.o.) postoperative.

### Intra- and postoperative complications

The intra- and postoperative complications are shown in Table 2. The intraoperative complication rate was 7.4% due to severe bleeding necessitating blood transfusion in 2 patients (Table 2A). The 30- and 90-day postoperative complication rate was 55.6% and 59.3%, respectively, with Clavien grade I and grade II being the main complication grades. Major complications were observed in 7.4% of the patients due to wound healing issues and/or wound infection, necessitating reintervention under general anaesthesia. We identified 12 grade I complications in 10 patients, 11 grade II complications in 8 patients, and 2 grade IIIb complications in 2 patients within the first 30 days postoperatively. One additional grade II complication occurred between day 30 and 90. An overview of the 30- and 90-day complications is given in Table 2B. The late complication rate was 14.8%. Hydronephrosis was the most common late complication, appearing in 3 (11.1%) patients. In all 3 patients the reason for hydronephrosis was surgery-related but, in only 1 case related to surgical reimplantation of the ureter (Table 2C).

**Table 2:** (**A**) Intraoperative complications, (**B**) postoperative 30-day and 90-day complications and (**C**) late complications (>90 days postoperatively) according to the Clavien-Dindo classification of surgical complications.

	Overall cohort (n = 27)
(**A**)	
**Intraoperative complications**	
**Intraoperative complication rate** (%)	2 (7.4)
Severe haemorrhage	2 (7.4)
**Intraoperative transfusion rate** (%)	2 (7.4)
(**B**)	
**Postoperative 30-day and 90-day complications**	
**30-day mortality rate** (%)	0 (0.0)
**Postoperative 30-day complication rate** (%)	15 (55.6)
**Rate of minor complications (**Clavien-Dindo 1–2)	13 (48.1)
**Rate of major complications** (Clavien-Dindo ≥3)	2 (7.4)
**Median Clavien-Dindo grade**	1 (0–2)
**Clavien-Dindo grade** (%)	
Grade 0	12 (44.4)
Grade I	10 (37.0)
Grade II	8 (29.6)
Grade IIIa	0 (0.0)
Grade IIIb	2 (7.4)
Grade IVa	0 (0.0)
Grade IVb	0 (0.0)
Grade V	0 (0.0)
**Number of complications**	
Grade 0	12
Grade I	12
Grade II	11
Grade IIIa	0
Grade IIIb	2
Grade IVa	0
Grade IVb	0
Grade V	0
**Kind of complications** (%)	
**Wound healing disorder**/**wound infections**	4 (14.8)
Grade 0	23 (85.2)
Grade I	1 (3.7)
Grade II	1 (3.7)
Grade IIIb	2 (7.4)
**Urine extravasation**/**Urinoma**	5 (18.5)
Grade 0	22 (81.5)
Grade I	2 (7.4
Grade II	3 (11.1)
**Urinary tract infection**	3 (11.1)
Grade 0	24 (88.9)
Grade II	3 (11.1)
**Dilatation of renal pelvis**	4 (14.8)
Grade 0	23 (85.2)
Grade I	4 (14.8)
**Subileus**	1 (3.7)
Grade 0	26 (96.3)
Grade II	1 (3.7)
**Lymphocele**	1 (3.7)
Grade 0	26 (96.3)
Grade I	1 (3.7)
**Cardiovascular events**^a^	3 (11.1)
Grade 0	24 (88.9)
Grade I	1 (3.7)
Grade II	2 (7.4)
**Others**^b^	5 (17.6)
Grade 0	22 (81.5)
Grade I	3 (11.1)
Grade II	2 (7.4)
**Postoperative transfusion rate** (%)	1 (3.7)
Grade 0	26 (96.3)
Grade II	1 (3.7)
**Postoperative 90-day complication rate** (%)	16 (59.3)
**90-day mortality rate (%)**	0 (0.0)
**Postoperative 30–90-day complication rate** (%)	1 (3.7)
**Rate of minor complications (**Clavien-Dindo 1–2)	1 (3.7)
**Rate of major complications** (Clavien-Dindo ≥3)	0 (0.0)
**Kind of complications** (%)	
**Lymphocele**	
Grade II	1 (3.7)
(**C**)	
**Late postoperative complications**	
**Late complication rate (>90 days postoperatively)** (%)	4 (14.8)
**Rate of minor complications (**Clavien-Dindo 1–2)	1 (3.7)
**Rate of major complications** (Clavien-Dindo ≥3)	3 (11.1)
**Clavien-Dindo grade** (%)	
Grade 0	23 (85.2)
Grade I	1 (3.7)
Grade II	0 (0.0)
Grade IIIa	0 (0.0)
Grade IIIb	3 (11.1)
Grade IVa	0 (0.0)
Grade IVb	0 (0.0)
Grade V	0 (0.0)
**Number of complications**	
Grade 0	23
Grade I	1
Grade II	0
Grade IIIa	0
Grade IIIb	4
Grade IVa	0
Grade IVb	0
Grade V	0
**Kind of complication** (%)	
**Surgery-related hydronephrosis**^**c**^	3 (11.1)
Grade 0	24 (88.9)
Grade IIIb	3 (11.1)
**Vesico-vaginal fistula**	1 (3.7)
Grade 0	26 (96.3)
Grade IIIb	1 (3.7)
**Chronic pain**	1 (3.7)
Grade 0	26 (96.3)
Grade I	1 (3.7)

^a^Tachyarrythmia (n = 2), tachycardia (n = 1).

^b^Acute urinary retention (n = 1), scrotum haematoma (n = 1), epididymitis (n = 1), lesion of the femoral nerve (n = 1), urinary bladder spasm (n = 1).

^c^Reasons for hydronephrosis: stenosis of reimplated ureter (n = 1), hydronephrosis due to scarred stenosis in the middle section of the ureter (n = 1), hydronephrosis due to pelvic lymphocele (n = 1).

### Oncological follow-up, adjuvant and palliative treatment, and outcomes

**Figure 1:**
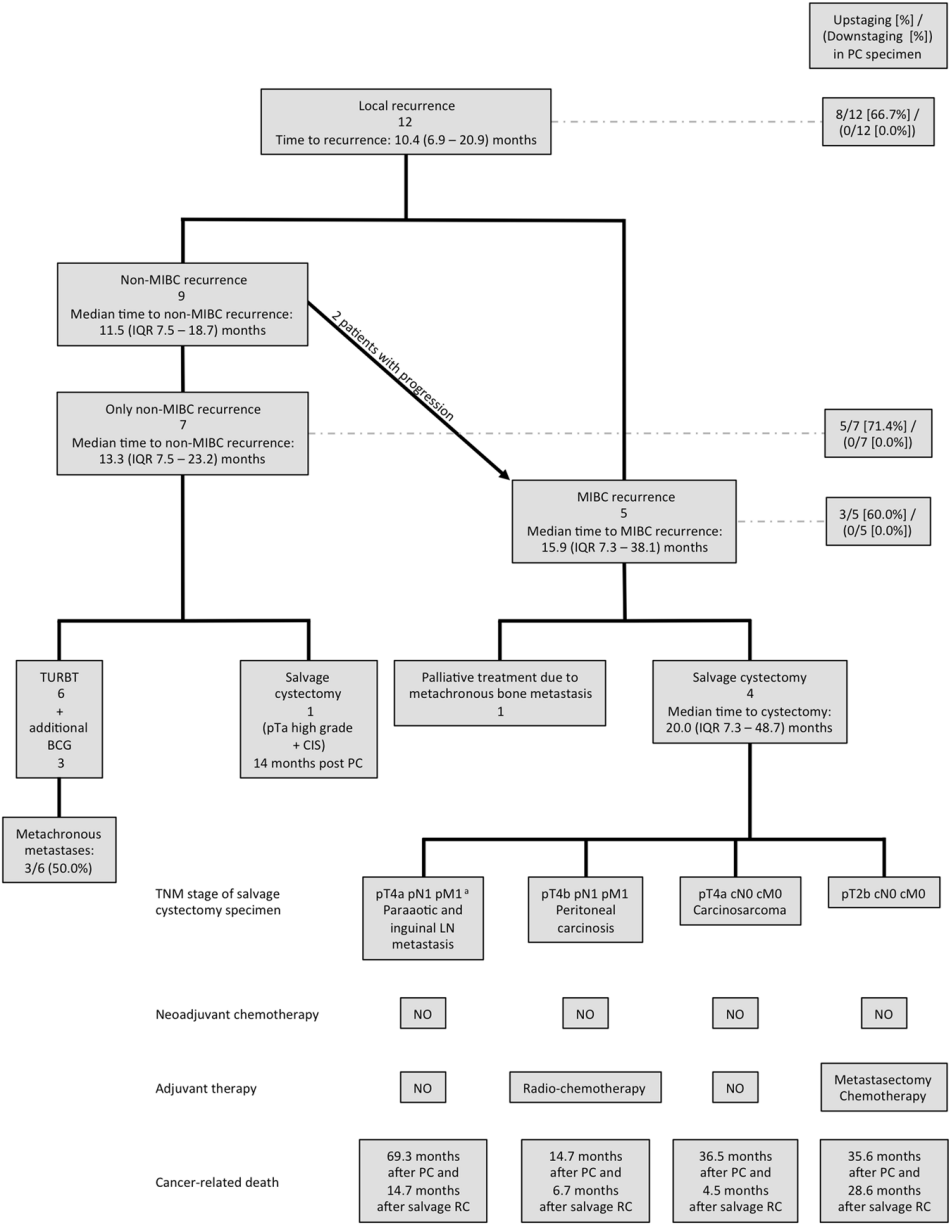
Local recurrence. Chart of patients with local recurrence after partial cystectomy (PC) for muscle-invasive bladder cancer (MIBC) showing number of patients affected, treatment modalities and oncological outcome. (MIBC) muscle-invasive bladder cancer, (PC) partial cystectomy, (IQR) inter quartile range, (TURBT) transurethral resection of bladder tumour, (BCG) Bacillus Calmette-Guerin, (CIS) carcinoma *in situ*, (LN) lymph node, (RC) radical cystectomy. ^a^Diagnosis of MIBC recurrence was 9.6 months before RC was performed.

The oncological follow-up, adjuvant and palliative treatments, and the outcome data are summarized in Table 3. Follow-up data was available for all patients. Overall, median follow-up time was 36.5 (23.3–78.8) months and 51.7 (33.9–90.9) months for survivors. Overall, 12 (44.4%) patients continued to be cancer-free, including all pT0 patients (18.5%), with a median disease-free time of 56.2 (36.1–90.9) months. Nine (36.0%) patients died from cancer, and the overall mortality rate was 55.5% (Table 3A). During the follow-up period, 22 (81.5%) patients preserved their bladder. Figure 1 shows a chart of patients who developed local tumour recurrence. Table 3B gives additional information on tumour stages in first local recurrence. There was no evidence of local tumour seeding with pelvic recurrences outside the bladder post PC. Altogether 10 (37.0%) patients developed metastasis after a median of 8.9 (4.8–27.7) months (Table 3C). Sixteen (59.3%) patients remained free from any progression. For the 11 (40.7%) patients who had tumour progression, the median progression free time was 36.1 (11.8–73.8) months (Table 3A). Patients with metastatic disease were treated with palliative systemic chemotherapy in 4 (14.8%) cases, with local radiation therapy in 2 (7.4%) cases, and with combined radiochemotherapy in 1 (3.7%) case. Three (11.1%) patients with metastases underwent additional local resection (Table 3D). The imaging performed during the course of the oncological follow-up is shown in Table 3E.

**Table 3:** Oncological follow-up after PC showing (**A**) the follow-up time and general oncological outcomes, (**B**) the incidence and time of local recurrence, (**C**) the incidence and time of distant metastasis, (**D**) adjuvant and post PC treatment, and (**E**) the imaging performed during the follow-up.

	Overall cohort (n = 27)
**(A)**	
**Follow-up times and oncological outcomes**	
**Follow-up time of overall cohort** (months)	36.5 (23.3 – 73.8)
**Minimum follow-up time of overall cohort** (months)	10.0
**Follow-up time of survivors** (months)	51.7 (33.9 – 90.9)
**Minimum follow-up time of survivors** (months)	20.1
**Complete disease-free** (%)	12 (44.4)
**Disease-free time** (months)	56.2 (36.1 – 90.9)
**MIBC and metastasis-free** (%)	16 (59.3)
**MIBC and metastasis-free time** (months)	51.9 (36.1 – 90.9)
**Progression-free** (%)	16 (59.3)
**Progression-free time** (months)	36.1 (11.8 – 73.8)
**Life-time bladder preservation** (%)	22 (81.5)
**Overall death rate** (%)	15 (55.5)
**Cancer-specific death rate** (%)	9 (36.0)
**(B)**	
**Local recurrence** (%)	12 (44.4)
**First local recurrence (%)**	
Non-MIBC	9 (33.3)
pTa	5 (18.5)
pT1	3 (11.1)
pTis	3 (11.1)
G1	0 (0.0)
G2	4 (14.8)
G3	4 (14.8)
GX	1 (3.7)
MIBC	3 (11.1)
**Local recurrence-free time** (months)	10.4 (6.9 – 20.9)
Time to non-MIBC recurrence	11.5 (7.5 – 18.7)
Time to MIBC recurrence	15.9 (7.3 – 38.1)
**Local recurrence-free (%)**	15 (55.5)
**Only non-MIBC in follow-up (%)**	7 (25.9)
**Progression from non-MIBC to MIBC in follow-up (%)**	2 (7.4)
**MIBC in follow-up (%)**	5 (18.5)
**(C)**	
**Distant metastasis** (%)	10 (37.0)
**Metastasis-free time** (months)	8.9 (4.8 – 27.7)
**Metastasis-free in follow-up (%)**	17 (63.0)
**(D)**	
**Post PC treatment**	
**Adjuvant treatment after PC (%)**	
**BCG intravesical instillation**	2 (7.4)
**Chemotherapy**	4 (14.8)
CMV	1 (3.7)
Gemcitabine/Cisplatin	2 (7.4)
Gemcitabine/Carboplatin	1 (3.7)
**Treatment of local recurrence (%)**	12 (44.4)
**Simple TURBT**	7 (25.9)
**Salvage cystectomy**	5 (18.5)
Time to salvage cystectomy from partial cystectomy (months)	14.0 (7.5 – 43.2)
at first local recurrence	3 (11.1)
organ-confined T stage of cystectomy specimen at first local recurrence	2 (7.4)
non-organ-confined T stage of cystectomy specimen at first local recurrence	1 (3.7)
at second local recurrence	2 (7.4)
non-organ-confined T stage of cystectomy specimen at second local recurrence	2 (7.4)
**BCG intravesical instillation**	3 (11.1)
**Treatment of metastatic disease (%)**	8 (29.6)
**Palliative chemotherapy**	4 (14.8)
Gemcitabine/Cisplatin	2 (7.4)
Gemcitabine mono	1 (3.7)
Unknown	1 (3.7)
**Metastasectomy**	3 (11.1)
**Combined radiochemotherapy**	1 (3.7)
**Radiation therapy**	2 (7.4)
**(E)**	
**Imaging rate** (%)	
Urethrocystoscopy	27 (100.0)
Ureterorenoscopy	0 (0.0)
CT/MRI of abdomen/pelvis	27 (100.0)
CT of thorax	13 (48.1)
X-ray of thorax	4 (14.8)
Excretion urogram	1 (3.7)
Bone szintigraphy	11 (40.7)
Sonography	27 (100.0)

Continous data are shown as median with interquartile range (IQR). (MIBC) muscle-invasive bladder cancer, (PC) partial cystectomy, (T) tumour, (CT) computed tomography, (MRI) magnetic resonance imaging (G) grading, (BCG) Bacillus-Calmette-Guérin, (TURBT) transurethral resection of bladder tumour, (CMV) Cisplatin, Methotrexat, Vinblastin.

### Survival rates

**Figure 2:**
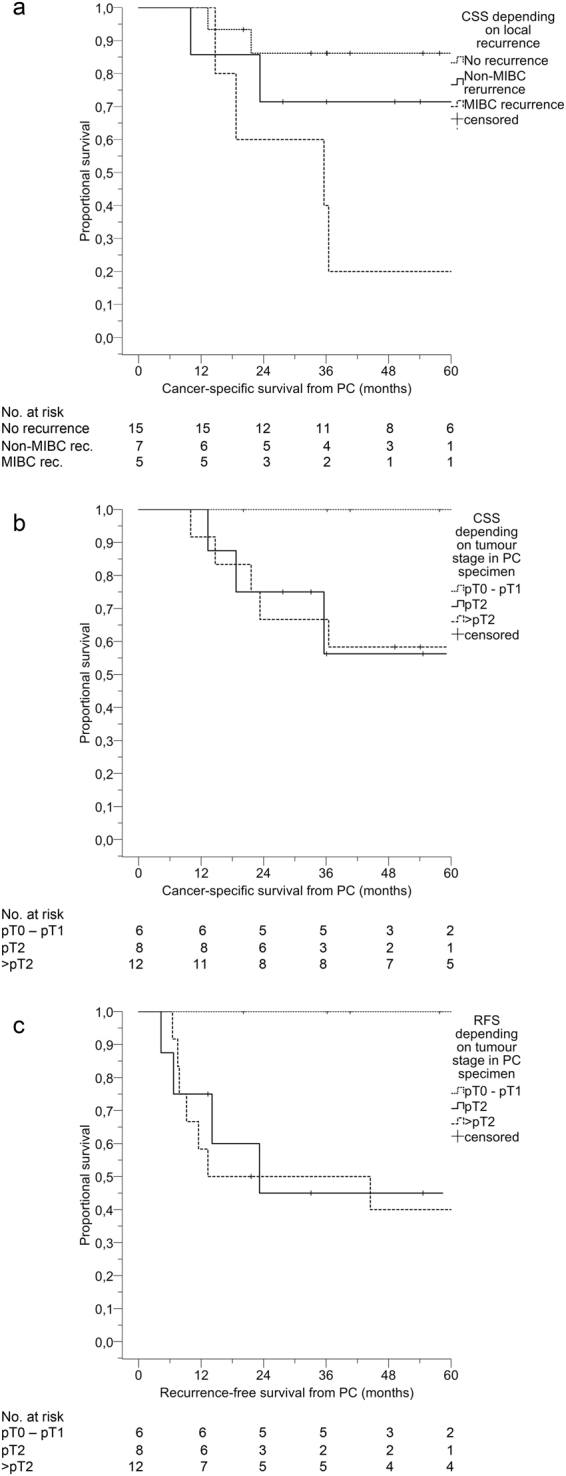
Kaplan-Meier plots. Plots for patients with MIBC treated with PC showing (**a**) CSS depending on local recurrence status (log rank test p = 0.004), (**b**) CSS depending on local tumour stage in PC specimen (log rank test p = 0.21), (**c**) RFS depending on local tumour stage in PC specimen (log rank test p = 0.09). (MIBC) muscle-invasive bladder cancer, (PC) partial cystectomy, (CSS) cancer-specific survival, (rec.) recurrence, (RFS) recurrence-free survival.

Estimated 5-year OS, CSS, local RFS, local MRFS, PFS, and MFS rates were 53.7%, 67.9%, 55.8%, 76.7%, 62.1%, and 63.3% respectively. In the subgroup analysis, estimated 5-year CSS was 86.2% for the subgroup without any local recurrence, 71.4% with local non-MIBC recurrence, and 20.0% with local MIBC recurrence, log rank p = 0.004 (Fig. 2a). Estimated 5-year CSS and local RFS were 100.0% (100.0%) for the subgroup staged pT0 – pT1 in the PC specimen, 56.3% (45.0%) for the subgroup staged pT2 in the PC specimen and 58.3% (40.0%) for the subgroup staged >pT2 in the PC specimen, log rank p = 0.21 (log rank p = 0.09) (Fig. 2b,c).

Simple Cox’s proportional hazards regression model identified MIBC tumour stage and vascular invasion of the PC specimen as well as the development of metachronous metastases to be significantly related to OS and CSS (Table 4A and B). Local recurrence of MIBC was found to constitute an additional risk factor for CSS (Table 4B) and borderline significance for OS (Table 4A). In addition, ureter reimplantation during PC was marginally significantly related to CSS. Variables significantly associated with PFS were MIBC tumour stage and vascular invasion of the PC specimen, multilocularity of the tumour, and reimplantation of the ureter during PC (Table 4C). Local RFS was significantly related to MIBC tumour stage of the PC specimen as well as to the time from primary diagnosis of BC to PC (Table 4D).

**Table 4:** Simple Cox’s proportional hazard ratio regression model investigating prognostic factors for (**A**) overall survival (OS), (**B**) cancer-specific survival (CSS), (**C**) progression-free survival (PFS), and (**D**) local recurrence-free survival (RFS).

A (Overall survival)	HR	95% CI	p-value
**Age** (years)	1.03	0.97–1.08	0.35
**Gender** m (ref.) vs. f	0.92	0.28–3.01	0.89
**Charlson comorbidity grade** (age adjusted) 1–2 (ref.) vs. 3–4	0.73	0.24–2.27	0.59
**Time of surgery** 1995–2005 (ref.) vs. 2006–2012	1.86	0.65–5.29	0.25
**History of Non-MIBC** No (ref.) vs. yes	0.54	0.19–1.54	0.25
**Time to PC from primary diagnosis of BC** (months)	1.01	0.99–1.03	0.34
**Time to PC from primary diagnosis of BC** <2 months (ref). vs. >2 months	1.13	0.38–3.33	0.83
**Lymph node status** pN0 (ref.) vs. pN+	0.67	0.21–2.20	0.67
**Number of LN dissected** (n)	1.00	0.90–1.12	0.97
**Number of LN dissected** ≤10 (ref.) vs. >10	1.10	0.34–3.54	0.88
**Extent of PLND** Unilateral (ref.) vs. bilateral	1.47	0.40–5.42	0.56
**Metastatic disease** No (ref.) vs. yes	5.78	1.91–17.50	0.002
**T stage of PC specimen** Non-MIBC (ref.) vs. MIBC	5.23	1.04–95.10	0.04
**Maximum tumour diameter** (cm)	1.24	0.88–1.74	0.22
**Lymphovascular invasion in PC specimen** No (ref.) vs. yes	2.67	0.87–8.23	0.09
**Vascular invasion in PC specimen** No (ref.) vs. yes	5.19	1.71–15.72	0.004
**Soft tissue margin of PC specimen** R0 (ref.) vs. R1	1.56	0.42–5.77	0.50
***CIS*** **in history or in PC** No (ref.) vs. yes	0.76	0.21–2.75	0.68
**Tumour extension** Unilocular (ref.) vs. multilocular	1.70	0.61–4.68	0.31
**Ureter reimplantation** No (ref.) vs. yes	2.42	0.83–7.08	0.12
**Local recurrence** No (ref.) vs. yes	2.19	0.77–6.23	0.14
**Local recurrence**			
No (ref.) vs. Non-MIBC	1.73	0.53–5.64	0.36
No (ref.) vs. MIBC	3.87	0.95–15.78	0.06
**B (Cancer-specific survival)**	**HR**	**95% CI**	**p-value**
**Age** (years)	0.99	0.93–1.06	0.86
**Gender** m (ref.) vs. f	1.20	0.30–4.85	0.80
**Charlson comorbidity grade** (age adjusted) 1–2 (ref.) vs. 3–4	0.47	0.12–1.74	0.26
**Time of surgery** 1995–2005 (ref.) vs. 2006–2012	1.19	0.29–4.86	0.81
**History of Non-MIBC** No (ref.) vs. yes	2.23	0.56–8.96	0.26
**Time to PC from primary diagnosis of BC** (months)	1.01	0.99–1.03	0.39
**Time to PC from primary diagnosis of BC** <2 months (ref). vs. >2 months	0.61	0.16–2.31	0.47
**Lymph node status** pN0 (ref.) vs. pN+	1.11	0.22–5.51	0.90
**Number of LN dissected** (n)	1.02	0.90–1.15	0.81
**Number of LN dissected** ≤10 (ref.) vs. >10	1.44	0.36–5.81	0.61
**Extent of PLND** Unilateral (ref.) vs. bilateral	0.74	0.18–3.10	0.68
**Metastatic disease** No (ref.) vs. yes	16.55	2.05–133.73	0.008
**T stage of PC specimen** Non-MIBC (ref.) vs. MIBC	307349623	1.65 – inf.	0.02
**Maximum tumour diameter** (cm)	1.31	0.83–2.08	0.25
**Lymphovascular invasion in PC specimen** No (ref.) vs. yes	2.48	0.59–10.47	0.22
**Vascular invasion in PC specimen** No (ref.) vs. yes	6.52	1.52–27.92	0.01
**Soft tissue margin of PC specimen** R0 (ref.) vs. R1	0.73	0.09–6.05	0.77
***CIS*** **in history or in PC** No (ref.) vs. yes	0.37	0.05–2.95	0.35
**Tumour extension** Unilocular (ref.) vs. multilocular	2.56	0.68–9.54	0.16
**Ureter reimplantation** No (ref.) vs. yes	3.72	0.98–14.13	0.05
**Local recurrence** No (ref.) vs. yes	5.41	1.12–26.20	0.04
**Local recurrence**			
No (ref.) vs. Non-MIBC	4.03	0.78–23.73	0.09
No (ref.) vs. MIBC	8.06	1.34–48.40	0.02
**C (Progression-free survival)**	**HR**	**95% CI**	**p-value**
**Age** (years)	0.94	0.86–1.03	0.18
**Gender** m (ref.) vs. f	1.82	0.30–10.97	0.51
**Charlson comorbidity grade** (age adjusted) 1–2 (ref.) vs. 3–4	0.36	0.06–2.19	0.36
**Time of surgery** 1995–2005 (ref.) vs. 2006–2012	1.01	0.17–6.11	0.99
**History of Non-MIBC** No (ref.) vs. yes	2.02	0.34–12.13	0.44
**Time to PC from primary diagnosis of BC** (months)	0.99	0.93–1.05	0.74
**Time to PC from primary diagnosis of BC** <2 months (ref). vs. >2 months	0.33	0.05–1.99	0.23
**Lymph node status** pN0 (ref.) vs. pN+	1.00	0.10–9.65	0.99
**Number of LN dissected** (n)	1.13	0.95–1.34	0.16
**Number of LN dissected** ≤10 (ref.) vs. >10	4.13	0.42–39.96	0.22
**Extent of PLND** Unilateral (ref.) vs. bilateral	1.23	0.13–11.91	0.86
**T stage of PC specimen** Non-MIBC (ref.) vs. MIBC	329839542	2.31 – inf.	0.005
**Maximum tumour diameter** (cm)	1.33	0.92–1.93	0.13
**Lymphovascular invasion in PC specimen** No (ref.) vs. yes	2.49	0.70–8.87	0.16
**Vascular invasion in PC specimen** No (ref.) vs. yes	5.33	1.49–19.09	0.01
**Soft tissue margin of PC specimen** R0 (ref.) vs. R1	1.10	0.23–5.21	0.90
***CIS*** **in history or in PC** No (ref.) vs. yes	0.29	0.04–2.25	0.24
**Tumour extension** Unilocular (ref.) vs. multilocular	4.53	1.32–15.53	0.02
**Ureter reimplantation** No (ref.) vs. yes	4.50	1.30–15.53	0.02
**D (Local recurrence-free survival)**	**HR**	**95% CI**	**p-value**
**Age** (years)	0.99	0.94–1.05	0.77
**Gender** m (ref.) vs. f	1.30	0.38–4.44	0.68
**Charlson comorbidity grade** (age adjusted) 1–2 (ref.) vs. 3–4	0.70	0.21–2.30	0.55
**Time of surgery** 1995–2005 (ref.) vs. 2006–2012	1.16	0.35–3.80	0.81
**History of Non-MIBC** No (ref.) vs. yes	1.74	0.52–5.80	0.37
**Time to PC from primary diagnosis of BC** (months)	1.03	1.01–1.05	0.01
**Time to PC from primary diagnosis of BC** <2 months (ref). vs. >2 months	0.79	0.25–2.50	0.69
**Lymph node status** pN0 (ref.) vs. pN+	0.72	0.16–3.35	0.68
**Number of LN dissected** (n)	1.02	0.91–1.14	0.72
**Number of LN** dissected ≤10 (ref.) vs. >10	1.29	0.37–4.48	0.69
**Extent of PLND** Unilateral (ref.) vs. bilateral	0.51	0.15–1.69	0.27
**T stage of PC specimen** Non-MIBC (ref.) vs. MIBC	335689225	2.60 – inf.	0.003
**Maximum tumour diameter** (cm)	1.22	0.87–1.72	0.25
**Lymphovascular invasion in PC specimen** No (ref.) vs. yes	1.13	0.24–5.24	0.88
**Vascular invasion in PC specimen** No (ref.) vs. yes	1.50	0.40–5.68	0.55
**Soft tissue margin of PC specimen** R0 (ref.) vs. R1	0.51	0.07–3.98	0.52
***CIS*** **in history or in PC** No (ref.) vs. yes	1.07	0.29–3.96	0.92
**Tumour extension** Unilocular (ref.) vs. multilocular	1.13	0.36–3.57	0.83
**Ureter reimplantation** No (ref.) vs. yes	1.87	0.58–6.00	0.30

(HR) hazard ratio, (CI) confidence interval, (m) male, (f) female, (ref.) reference, (vs.) versus, (MIBC) muscle-invasive bladder cancer, (PC) partial cystectomy, (BC) bladder cancer, n (number), (LN) lymph node, (PLND) pelvic lymphadenectomy, (T) tumour, (*CIS*) carcinoma *in situ*, (inf.) infinity.

### Health-related quality of life (HR-QoL) data (EORTC QLQ-C30)

Global health status/QoL scale was 79.2 (52.1–97.9) for PC, 66.7 (50–83.3) for RC (all forms of urinary diversion), 58.3 (33.3–83.3) for RC and urinary diversion with ileal conduit, and 70.8 (56.2–85.4) for RC and urinary diversion with orthotopic ileal neobladder. The results for multi-item function scales, multi-item symptom scales, and single-item scales of the EORTC QLQ-C30 questionnaire in patients treated with PC or RC are summarized in Table 5A–D.

**Table 5:** Comparison of health-related quality of life (HR-QoL) data assesed by EORTC QLQ-C30 questionnaire in patients treated with (**A**) partial cystectomy or (**B**) radical cystectomy (both types of urinary diversion included – ileal conduit and orthotopic ileal neobladder) and in the subroup of radical cystectomy patients treated with (**C**) ileal conduit or (**D**) orthotopic ileal neobladder.

EORTC QLQ-C30 (version 3.0)	A	B	C	D
	Partial cystectomy Mean ± SD Median (IQR) n = 12	Radical cystectomy^a^ Mean ± SD Median (IQR) n = 58	Ileal conduit^a^ Mean ± SD Median (IQR) n = 24	Ileal neobladder^a^ Mean ± SD Median (IQR) n = 34
Global health status/quality of life scale	75.0 ± 23.0	66.5 ± 23.0	58.0 ± 25.3	72.3 ± 19.5
	79.2 (52.1–97.9)	66.7 (50.0–83.3)	58.3 (33.3–83.3)	70.8 (56.2–85.4)
		n = 57	n = 23	n = 34
**Multi-item function scales**				
Physical functioning	81.1 ± 10.6	75.7 ± 25.5	65.8 ± 29.4	82.6 ± 19.9
	80.0 (73.3–86.7)	86.7 (53.3–100.0)	70.0 (33.3–93.3)	93.3 (71.6–100.0)
Role functioning	68.1 ± 29.7	71.0 ± 29.2	63.8 ± 31.1	76.0 ± 27.9
	66.7 (37.5–100.0)	75.0 (50.0–100.0)	66.7 (33.3–100.0)	83.3 (50.0–100.0)
Emotional functioning	61.1 ± 30.4	77.4 ± 22.6	72.2 ± 22.3	81.1 ± 22.3
	70.8 (33.3–83.3)	83.3 (58.3–100.0)	70.8 (52.1–91.7)	87.5 (66.7–100.0)
Cognitive functioning	86.1 ± 26.4	81.0 ± 21.5	77.8 ± 22.9	83.3 ± 20.5
	100.0 (83.3–100.0)	83.3 (66.7–100.0)	83.3 (54.2–100.0)	83.3 (66.7–100.0)
Social functioning	80.6 ± 26.4	68.1 ± 32.5	65.3 ± 32.2	70.1 ± 33.0
	100.0 (66.7–100.0)	83.3 (33.3–100.0)	83.3 (33.3–95.8)	75.0 (45.8–100.0)
**Multi-item symptom scales**				
Fatigue	20.4 ± 17.6	30.8 ± 28.5	37.5 ± 28.1	26.0 ± 28.3
	22.2 (0.0–33.3)	33.3 (0.0–55.6)	33.3 (11.1–63.9)	16.7 (0.0–55.6)
Nausea and vomiting	8.3 ± 20.7	6.0 ± 16.4	9.7 ± 20.2	3.4 ± 12.8
	0.0 (0.0–0.0)	0.0 (0.0–0.0)	0.0 (0.0–12.5)	0.0 (0.0–0.0)
Pain	13.9 ± 21.1	21.8 ± 33.1	26.4 ± 31.8	18.6 ± 34.0
	0.0 (0.0–33.3)	0.0 (0.0–33.3)	8.3 (0.0–50.0)	0.0 (0.0–33.3)
**Single-item symptom scales**				
Dyspnoea	13.9 ± 22.3	31.6 ± 36.6	37.5 ± 35.9	27.5 ± 37.1
	0.0 (0.0–33.3)	33.3 (0.0–66.7)	33.0 (0.0–66.7)	0.0 (0.0–41.7)
Insomnia	33.3 ± 40.2	24.7 ± 29.0	29.2 ± 31.6	21.6 ± 27.1
	16.7 (0.0–66.7)	16.7 (0.0–33.3)	33.3 (0.0–66.7)	0.0 (0.0–33.3)
Appetite loss	5.6 ± 19.5	11.5 ± 24.6	18.1 ± 31.1	6.9 ± 17.9
	0.0 (0.0–0.0)	0.0 (0.0–0.0)	0.0 (0.0–33.3)	0.0 (0.0–0.0)
Constipation	8.3 ± 20.7	16.9 ± 25.2	22.2 ± 30.6	11.8 ± 19.9
	0.0 (0.0–0.0)	0.0 (0.0–33.3)	0.0 (0.0–33.3)	0.0 (0.0–33.3)
Diarrhoea	2.8 ± 9.6	15.5 ± 27.4	4.2 ± 14.9	23.5 ± 31.3
	0.0 (0.0–0.0)	0.0 (0.0–33.3)	0.0 (0.0–0.0)	0.0 (0.0–33.3)
Financial difficulties	2.8 ± 9.6	22.4 ± 33.9	25.0 ± 35.8	20.6 ± 32.8
	0.0 (0.0–0.0)	0.0 (0.0–66.7)	0.0 (0.0–66.7)	0.0 (0.0–41.7)

Continuous data are shown as mean with standard deviation (SD) and as median with interquartile range (IQR).

EORTC QLQ-C30: the questionnaire assesses cancer-specific quality-of live data. In all questions a scale from 1 to 4 was used (1: not at all, 2: a little, 3: quite a bit, 4: very much). All scales were linearly transformed such that all scales range from 0 to 100. For functional and overall scales higher scores represent a better outcome on quality of life (QoL), whereas for symptom and single-item scales higher scores correspond to more problems and a reduced QoL.

^a^Data previously published by Erber *et al*. Morbidity and Quality of Life in Bladder Cancer Patients following Cystectomy and Urinary Diversion: A Single-Institution Comparison of Ileal Conduit versus Orthotopic Neobladder, Urol 2012;2012:342796.

## Discussion

Historical PC series published in the 1970’s reported high rates of local recurrence and poor oncological outcome^18–21^. However, PC has experienced some resurgence as a less morbid and oncologically effective treatment^6^. With more stringent selection criteria that limits PC to patients with a solitary primary MIBC-tumour, without concomitant CIS or a history of non-MIBC, and feasible for full-wall resection with an adequate safety margin, 5-year OS rates of 53–70% and RFS rates of 40–64% have been reported in more recent series^7–10,22–24^. However, it is estimated that only 3–10% of patients with MIBC meet these criteria^9,25–27^. During the last ten years, the utilization rate of PC has remained stable in the United States of America, accounting for 7%-10% of all cystectomies performed^28^. European data on PC rates is currently limited. Taking into account that only 52.5% of patients with MIBC received aggressive treatment therapy in the series (2004–2008) by Gray *et al*.^28^, only 2.8% of patients with MIBC underwent a PC. In this series, elderly patients aged 81–90 years were the group that had the lowest rate of aggressive treatment therapies, with only 35% receiving aggressive therapy defined as RC or PC or definitive radiotherapy (RT)/chemoradiotherapy. It is this group that might benefit the most from a less morbid approach. Thus, these numbers suggest that in general, all aggressive therapies for BC including PC are underutilized, although older data (1988–2000) from the Surveillance, Epidemiology and End Results (SEER) registry and the Nationwide Inpatient Sample (NIS) showed a PC rate of 13–17% disproportionately used in certain medical centres (nonteaching, rural, low volume) and patient populations (elderly, black, females, stage I disease) reflecting selective referral or overuse^27^.

However, in our series PC was mainly performed in patients with a high Charlson Comorbidity Grade or ACE-27 Score, taking into consideration that complications related to RC may be directly related to pre-existing comorbidity as well as to bowel anastomosis or urinary diversion^3–5^, and that comorbidity was identified to be an independent prognostic factor for perioperative mortality, overall mortality, and cancer-specific mortality in RC patients^3–5^. Thus, the selection of patients in our series differed from the stringent selection criteria for PC since patients with multifocal disease, the necessity to reimplant the ureter, and a prior history of non-MIBC were included. However, we showed 5-year OS, CSS, local RFS, and PFS rates of 53.7%, 67.9%, 55.8%, and 62.1%. This is consistent with PC series published by Knoedler *et al*.^7^, Zhang *et al*.^29^, and Ma *et al*.^23^, but worse in comparison to cohorts published by Kassouf *et al*.^9^, Holzbeierlein *et al*.^8^, Smaldone *et al*.^24^, or Koga *et al*.^30^ (Table 6). These differences may reflect differences in patient selection, patient numbers, age, extend of pelvic lymph node dissection, and impact of neo-adjuvant or adjuvant treatment. In addition, definitions of RFS are inconsistent and include also distant metastases in some publications. Furthermore, Koga’s series^30^ is the only one based on a very stringent bladder-sparing protocol consisting of neo-adjuvant low-dose radiochemotherapy (NARC) followed by PC and PLND with well-defined inclusion criteria for PC. Knoedler’s series^7^ is the only one also reporting comorbidity data, showing that severe comorbidities are associated with an impaired OS and CSS.

**Table 6:** Fife-year oncological outcomes in different PC series for the treatment of muscle-invasive bladder-cancer.

Reference	Year of publication	Number of PC patients	Age	OS	CSS	RFS
Ebbing *et al*.	2018	27	Median 66.0 (IQR 56.0–76.0)	53.7.0%	67.9%	55.8%^1^
Knoedler *et al*.^7^	2012	86	Median 68.5 (IQR 50.9–72.3)	53.0%	65.0%	No data
Zhang *et al*.^29^	2010	100	Mean 65.7 (SD ± 12.7)	No data	68.0%	29.0%^1^
Ma *et al*.^23^	2013	101	Median 69.0 (range 24.0–84.0)	58.0%	65.0%	50.0%^2^
Kassouf *et al*.^9^	2006	37	Median 69.9 (range 40.0–82.0)	67.0%	87.0%	39.9%^2^
Holzbeierlein *et al*.^8^	2004	58	Mean 65.5 (range 16.0–91.0)	69.0%	No data	No data
Smaldone *et al*.^24^	2008	25	Mean 65.1 (SD ± 9.8)	70.0%	84.0%	64.0%^2^
Koga *et al*.^30^	2011	46	Median 74.0 (IQR 63.0–81.0)	No data	100.0%	77.0%^1^

(OS) overall survival, (CSS) cancer-specific survival, (RFS) recurrence-free survival, (IQR) interquartile range, (SD) standard deviation.

^1^Recurrence defined as local recurrence within the bladder (non-muscle invasive and/or muscle-invasive).

^2^Recurrence defined as local recurrence within the bladder (non-muscle invasive and/or muscle-invasive) and/or development of pelvic or distant metastasis.

Differences in selected patient cohorts between the series make comparisons in oncological outcomes challenging, however some conclusions might be drawn. Despite a higher rate of node positive disease in our cohort (22.2%) compared to the series of Knoedler *et al*. (11%)^7^, Zhang *et al*. (5%)^29^, Kassouf *et al*. (14%)^9^, and Smaldone *et al*. (12%)^24^, adjuvant chemotherapy was given more often in Kassouf’s (24%), Zhang’s (21% adjuvant chemoradiotherapy), or Smaldone’s (40%) cohort compared to our (14.8%) or Knoedler’s cohort (5.8%). In addition, in Smaldone’s series^24^ all patients were treated with neoadjuvant local radiation to the abdominal wall and adjuvant intravesical BCG instillation therapy for six weeks, which might reduce the risk of local recurrence. Remarkably, Smaldone’s^24^ and Koga’s^30^ patient cohorts showed the highest CSS (84%^24^ and 100%^30^) and RFS (64%^24^ and 77%^30^) rates of the discussed series, but positive pelvic lymph node involvement was much lower (12%^24^ and 0%^30^ vs. 22.2% in our cohort). Of note, patients in Koga’s series^30^ treated with a completed protocol including PC after NARC developed only superficial local recurrence and 5-year MFS was 100% while 23.1% of patients treated with the same protocol but declined PC developed local MIBC recurrence, and 5-year MFS was significantly reduced to 83%.

Ma *et al*.^23^ and Zhang *et al*.^29^ concluded that lymphovascular invasion was a risk factor for poor prognosis after PC. Holzbeierlein *et al*.^8^, Ma *et al*.^23^, Zhang *et al*.^29^, and Koga *et al*.^30^ identified multifocality to be associated with local recurrence. Kassouf *et al*.^9^ and Ma *et al*.^23^ reported a history of prior cancer to also be a risk factor for tumour recurrence and impaired survival. In addition, Ma *et al*.^23^ identified ureter reimplatation to be associated with poor prognosis. Despite our less stringent patient selection, we also identified ureter reimplantation, vascular invasion, and tumour multilocularity to be risk factors for tumour progression. In the 3 series^8,9,24^ that showed better survival outcomes, ureter reimplantation was absent in all and tumour multifocaltiy in two^9,24^. In Koga’s cohort^30^, ureter reimplantation (26%) was also performed but not included in the regression analysis; however, this cohort showed no local MIBC recurrence. Notably, in Zhang’s cohort^29^, ureter reimplantation (22%) was associated with a higher CSS rate, but the authors recommend to consider this “unreasonable” result cautiously. Eventually, the need for ureter reimplantation and a multifocality of the tumour should be regarded as comparative contraindications.

However, survival data after PC has been shown to compare favourably with RC series^7,10^ or series after a trimodal therapy (TMT) approach, including maximal TURBT followed by concurrent radiosensitising chemotherapy and RT^31^. A TMT approach is the most studied bladder-sparing strategy, and it is considered a valuable alternative treatment for selected patients with T2 MIBC^2^. However, in our institution, we estimate that only 3–5 patients undergo TMT per year, compared to about 60 RCs per year. Overall, the 5-year CSS ranges from 50% to 82% and the 5-year OS is approximately 50%, ranging from 36% to 74%. The local recurrence rate after TMT ranges from 24% to 43%. Of these recurrent tumours, the rate of MIBC recurrences ranges from 11% to 18.5%, resulting in an average overall salvage cystectomy rate of approximately 25–30%. Overall, the rate of RC for late effects of RT ranges from 0% to 2%^31^.

The 5-year CSS and OS rates after RC are approximately 62%–66%^7,10,32–34^. RFS is roughly 58–68% after RC, but varies between 34% and 74% depending on local tumour stage and nodal status^33,34^. Two matched analyses from Capitanio *et al*.^10^ and Knoedler *et al*.^7^ comparing PC with RC demonstrated no statistically significant differences in cancer control, neither in CSS nor in OS rates. Nevertheless, patients treated with PC remain at risk for intravesical local recurrence. In our cohort, 55.5% of patients remained free of local recurrence, 18.5% developed local MIBC recurrence, the rest superficial recurrence, resulting in a salvage cystectomy rate of 18.5%. Notably, 2 of the 12 patients who experienced local recurrence in our study developed late local MIBC recurrence 32 and 45 months after PC, emphasizing the importance of a life-long surveillance after PC. The aforementioned publications showed similar results with local MIBC recurrences ranging between 8 and 31%, superficial recurrences between 8 and 24%^8,9,23,24^, and a rate of late cystectomies of 7–19%^7–9,23,24^.

In our series, major 30 to 90-day complications occurred in 7.4%, and major late complications in 11.1% of patients, with wound healing disorder or wound infections constituting the most common short-term and hydronephrosis the most common long-term complications requiring intervention. Little data currently exists regarding complications of PC. To our knowledge, we are the first group that uses the Clavien-Dindo classification to describe complications in an open PC series. Smaldone *et al*.^24^ and Holzbeierlein *et al*.^8^ rudimentarily describe postoperative complications in their open PC cohorts. In Smaldone’s study^24^, a long-term rate of distal ureter stenosis requiring ureteroneocystostomy was observed in 4% of cases. Kates *et al*.^35^ utilized the Nationwide Inpatient Sample (NIS) to examine 10780 patients who underwent PC for BC. They found that 15.8% of patients experienced an in-hospital complication and 3.9% a “never event” -in-hospital complication with a mortality rate of 1.8%. RC series which used the Clavien-Dindo scoring describe a rate of 13–36% major (Clavien-Dindo ≥3) 90-day complications depending on age, surgical technique, and type of urinary diversion investigated^36–41^.

The only other PC study group (Golombos *et al*.^42^) using the Clavien-Dindo score in a robot-assisted PC approach (also including non-MIBC) describes an overall 90-day complication rate of 24.1% with no occurrence of major complications.

In the past years, there has been an increasing interest on QoL outcomes in urological malignancies, developing new specific instruments with the aim of evaluating the HR-QoL of the patients and the impact of the respective health condition on their lives. Nevertheless, QoL outcomes are difficult to compare. Many different questionnaires are used to measure the QoL in oncological urology (e.g. SF-36 (Short Form-36), FACT-G (Functional Assessment of Cancer Therapy - General), EORTC QLQ-C30); the questionnaires differ considerably in some of the topics covered^43,44^. Another difficulty is that QoL is a multi-dimensional concept, incorporating many domains that are weighted by their importance to the individual and may change over time^45,46^. In addition, continent and incontinent forms of urinary diversion after radical surgery for bladder cancer were shown to influence postoperative QoL with different impact, eventually favouring orthotopic neobladder, but consistent evidence from systematic reviews and meta-analyses is lacking^43,44^. An interaction could even be demonstrated between patients and the investigating institution which has hypothetically been attributed to glorification and idealization of the attending urologists^47^. Thus, we used EORTC QLC-C30 QoL data from patients treated with RC at our institution and faced the data with this PC series to minimize confounding factors and to achieve high comparability. Compared to patients treated with RC and orthotopic ileal neobladder, in our institution postoperative global health status and QoL outcomes showed equally high scores in this series of patients treated with PC but higher scores compared to patients treated with RC and ileal conduit. This result might be due to the surgical procedures, resulting in continent or incontinent urinary conditions. On the other hand, patients following PC suffered more often from emotional problems compared to patients after RC and orthotopic ileal neobladder. However, patients following PC experienced financial difficulties caused by their physical condition or medical treatment less frequently compared to patients following RC. This difference was most obvious after urinary diversion with a conduit. However, this observation has to be interpreted with caution since the number of cases is small in each group. In addition, the three groups differ in terms of age, gender distribution, and comorbidity grade. Patients provided with orthotopic ileal neobladder were a median four years younger (62 [56–66]) and patients with ileal conduit four years older (70 [64–75]) compared to patients treated with PC. Patients following PC or RC with ileal conduit had higher Charlson comorbidity grades at time of surgery (3 [2–4]; 3 [2–3]) than patients after RC with orthotopic ileal neobladder (2 [2–3]), and 96% of the patients treated with RC and orthotopic ileal neobladder were males compared to 70% in the PC series. Hence, a statistically robust comparison is ineligible, and firm conclusions cannot be drawn. Furthermore, since no baseline QoL data were available, a comparison of pre- and postoperative scores was not possible.

Considering external data, the systematic review and meta-analysis by Yang *et al*.^44^ showed a median global health status/QoL scale in the EORTC QLQ-C30 questionnaire of 72 for RC with continent urinary diversion, and of 65 for RC with incontinent urinary diversion, which is in line with our presented data.

Although aiming to help to improve QoL if the bladder function remains preserved after treatment, QoL data after TMT are very limited and are mainly focused on late toxicity. Data comparing QoL after PC and TMT in patients with MIBC are lacking. However, better QoL was shown after TMT compared to RC with incontinent urinary diversion when using a self-administered questionnaire^48^.

Our study is limited in its retrospective nature and small sample size. However, we confirmed that PC can provide adequate cancer control of MIBC in selected cases and showed that it offers patients a good chance of a long-term bladder preservation, providing a good postoperative global health status and QoL and that it is associated with only a moderate rate of severe perioperative complications, even in comorbid patients. Though, survival data of our presented study and of the cited PC series refer to selected patient cohorts, making a direct comparison to RC or TMT series difficult.

However, the outcome of MIBC is determined more by the extent of disease (stage) than by the extent (type) of surgery. Hence, to cure MIBC, removal of the tumour with negative surgical margins and no residual tumour is required, regardless of the procedure used^49^. That might also include an en-bloc tumour resection in the previous transurethral staging operation to better define the margin during PC, but this approach is still considered experimental^50,51^.

In addition, there is no doubt that neoadjuvant chemotherapy (NAC) improves survival of MIBC over RC alone^52–54^. Thus, although proven for RC, NAC should also be considered in MIBC patients who are candidates for PC and suitable for NAC. Since patients who decide to undergo PC are likely also more willing to take the risk of under-treatment (rather than the risk of side effects or restrictions in QoL), it is not surprising that none of our PC patients was treated with neoadjuvant chemotherapy, even though we offered NAC to suitable candidates. This stresses the fact that PC cohorts, including our series, represent highly selected cases.

In conclusion, patient selection with clearly defined inclusion and exclusion criteria are essential to further improve oncological outcomes of PC and MIBC patients in general.

We need prospective randomized controlled trials (RCT) in MIBC comparing RC with PC and TMT in the subgroup of patients potentially suitable for a bladder-sparing therapy protocol with strictly defined inclusion criteria (e.g. primary, small, solitary tumours with response to NAC, including pre-therapeutic genetic tests of the tumour heterogeneity to predict the response to NAC und to increase the acceptance of NAC). This could clarify the essential oncological and quality of life endpoints between the different therapies and could answer the question who is likely to benefit from a less invasive approach and for which patient a RC might be an unnecessary overtreatment. Unfortunately, previous attempts of RCTs, like the SPARE (Selective Bladder Preservation Against Radical Excision) trial, which compares outcomes in patients who receive NAC followed by RC or RT, failed to recruit patients. Strong clinician and patient preferences for treatments impacted willingness to undergo randomization and acceptance of treatment allocation^55^, which underlines the fact that high patients’ preferences for specific treatment options is not only a bias of retrospective studies, but also impacts the design and success of RCTs.
